# Recombination events among virulence genes in malaria parasites are associated with G-quadruplex-forming DNA motifs

**DOI:** 10.1186/s12864-016-3183-3

**Published:** 2016-11-03

**Authors:** Adam Stanton, Lynne M. Harris, Gemma Graham, Catherine J. Merrick

**Affiliations:** 1 School of Computing and Mathematics, Faculty of Natural Sciences, Keele University, Keele, Staffordshire ST55BG UK; 2 Centre for Applied Entomology and Parasitology, Faculty of Natural Sciences, Keele University, Keele, Staffordshire ST55BG UK; 3 School of Medicine, Keele University, Keele, Staffordshire ST55BG UK

**Keywords:** *Plasmodium*, Malaria, G quadruplex, *Var* genes, Recombination

## Abstract

**Background:**

Malaria parasites of the genus *Plasmodium* possess large hyper-variable families of antigen-encoding genes. These are often variantly-expressed and are major virulence factors for immune evasion and the maintenance of chronic infections. Recombination and diversification of these gene families occurs readily, and may be promoted by G-quadruplex (G4) DNA motifs within and close to the variant genes. G4s have been shown to cause replication fork stalling, DNA breakage and recombination in model systems, but these motifs remain largely unstudied in *Plasmodium*.

**Results:**

We examined the nature and distribution of putative G4-forming sequences in multiple *Plasmodium* genomes, finding that their co-distribution with variant gene families is conserved across different *Plasmodium* species that have different types of variant gene families. In *P. falciparum*, where a large set of recombination events that occurred over time in cultured parasites has been mapped, we found a strong spatial association between these recombination events and putative G4-forming sequences. Finally, we searched *Plasmodium* genomes for the three classes of helicase that can unwind G4s: *Plasmodium spp.* have no identifiable homologue of the highly efficient G4 helicase PIF1, but they do encode two putative RecQ helicases and one homologue of the RAD3-family helicase FANCJ.

**Conclusions:**

Our analyses, conducted at the whole-genome level in multiple species of *Plasmodium*, support the concept that G4s are likely to be involved in recombination and diversification of antigen-encoding gene families in this important protozoan pathogen.

**Electronic supplementary material:**

The online version of this article (doi:10.1186/s12864-016-3183-3) contains supplementary material, which is available to authorized users.

## Background

Human malaria gives rise to widespread morbidity and more than half a million deaths each year [1]. It is caused by protozoan *Plasmodium* parasites, with most of the mortality being due to the species *Plasmodium falciparum*. These parasites cause illness via the cyclical infection of erythrocytes. They multiply inside these cells and modify their surfaces with proteins called *P. falciparum* Erythrocyte Membrane Protein 1 (PfEMP1) [2], which bind to the walls of blood vessels. PfEMP1 proteins are crucial virulence factors, preventing infected cells from circulating through and being destroyed by the spleen, but also contributing to disease [3, 4]. Severe malaria is particularly associated with sequestration of infected cells in vessels of the brain and placenta.

To prevent the human immune system from recognizing parasite proteins exposed on infected erythrocytes, *P. falciparum* regularly switches to express different PfEMP1 variants. It possesses a large family of genes called *var* that encode these proteins [2, 5, 6], and varies their expression by epigenetic silencing and switching [7]. The parasite can thus evade immunity and sustain a chronic infection for months or years [8–10], ensuring its transmission to new hosts. Furthermore, *var* genes recombine readily to generate new variants [11–13] - both during meiosis, when sexual reproduction occurs in the gut of the mosquito vector [14], and also during mitosis. The parasite is haploid in all non-sexual stages of its lifecycle but inter-allelic recombination can still generate new *var* gene variants from a single haploid genome [11, 12]. Thus, the numerous parasite strains that circulate in endemic regions all have unique repertoires of virulence genes [15] and this is one reason why immunity to repeated malaria infections is slow to develop in humans.

Being a major virulence factor in malaria, *var* gene biology has been extensively studied, establishing that both meiotic and mitotic recombination can generate *var* gene diversity, and also that antigenic variation occurs via epigenetic silencing and switching of *var* gene expression. Silencing is facilitated by the location of most of the *var* genes in heterochromatic subtelomeric regions of the genome; in fact, the remaining subset of *var* genes found in chromosome-internal tandem arrays is likewise heterochromatinized [16]. Nevertheless, the molecular mechanisms that control both recombination and silencing/switching are not yet fully understood. A role for G-quadruplex (G4) DNA motifs has been proposed [17, 18], but not yet experimentally tested.

G-quadruplex motifs can form from DNA sequences that contain four closely-spaced tracts of at least three guanines, separated by short tracts of other nucleotides [19]. Such sequences have been extensively studied in yeast and human cells, proving that they can form intra-molecular quadruplex structures both in vitro and in vivo [20]. These unusual structures interrupt the normal double-helical structure of DNA and have important biological roles: they regulate telomere structure [21], inhibit gene transcription [22] and can promote recombination via stalling of replicative polymerases [23, 24]. Indeed, drugs that specifically bind to and stabilize G4s can further inhibit transcription and also predispose DNA to replicative instability [25, 26]. Furthermore, in the pathogenic bacterium *Neisseria gonorrhoeae*, antigenic switching amongst pilin proteins is initiated by a G4 motif upstream of the expression site of the *pilE* virulence gene [27].

It is therefore possible that G4 motifs could affect the silencing, expression switching and recombination of *var* genes in *P. falciparum*. A bioinformatic analysis of the *P. falciparum* genome has shown that there are remarkably few putative quadruplex sequences (PQSs) in this genome, which is one of the most highly A/T biased genomes ever sequenced at ~81 % A/T [28]. The great majority of PQSs are found in the telomeres because *Plasmodium* telomeres, as in other organisms, consist of a guanine-rich repeat that is intrinsically prone to form G4s. Only 63 PQSs were found in this genome outside of the telomeres, even when a relatively relaxed prediction algorithm was used [17]. 31 of the 63 non-telomeric PQSs were within or upstream of *var* genes, and biophysical methods confirmed that a selection of these sequences could adopt G4 conformations in vitro under physiological conditions. The strong association between PQSs and *var* genes in this unusually G4-poor genome is very suggestive of a role for these sequences in virulence gene control.

G4 motifs can take up various structures depending on factors such as the alignment of the guanine-containing strands, the number of strands involved and the lengths of the nucleotide loops separating the runs of guanines [29]. Different G4 structures have different stabilities in vitro and in a yeast model system it was recently shown that this relates to their ability to promote recombination in vivo. Highly stable G4s, which generally featured very small loops containing single pyrimidines rather than single purines, were particularly prone to cause recombination events [30]. The authors then went on to establish that such sequences were under-represented in several other genomes from *Caenorhabditis elegans* to human, indicating that their particular recombinogenic properties may be selected against during genome evolution [30].

Here we explore several questions concerning G4 motifs in *Plasmodium* genomes. Firstly, are the PQSs in the *P. falciparum* genome likely to promote recombination, and might the ones associated with *var* genes be more recombinogenic than those elsewhere in the genome? We hypothesize that if genome-destabilizing PQSs are generally selected against, these sequences should be under-represented, as they are in other genomes [30]. However, if *var*-associated PQSs can play a specific positive role in promoting inter-*var*-gene shuffling, conferring a potential advantage in immune evasion, then counter-selection might occur for high recombinogenic potential amongst *var*-associated PQSs. An analogous theory has been proposed to explain the presence of the unique short-loop G4 motif that is found upstream of the *pilE* locus in *N. gonorrhoeae* [30].

Secondly, moving from theoretical analysis to experimental data, we examine whether PQSs actually associate with recombination breakpoints detected in vivo in the *P. falciparum* genome. Two recent studies have mapped a large number of mitotic recombination breakpoints occurring in parasites cultured for long periods, and these occurred primarily in regions that encode *var* genes [11, 12]. One possible mechanism for the recombination events would be a stalled replication fork caused by a G4, requiring repair via recombination with another *var* gene sequence.

Thirdly, we investigate whether it is a general feature of *Plasmodium* species that PQSs are associated with variantly-expressed virulence gene families, or whether this is unique to *P. falciparum*. The *var* gene family itself occurs only in *P. falciparum* and closely related ape parasites [31]. Other *Plasmodium* species that do not encode *var*s possess large families of ‘*pir*’ genes, which are also variantly expressed and may play analogous roles in antigenic variation and immune evasion [32, 33].

Finally, we examine the prospects for G4 metabolism in *Plasmodium* parasites by searching the genomes for helicases that specifically unwind G4 motifs.

We show that PQS motifs with high predicted stability do not clearly over-associate with *var* genes, but nor are they selected against in the *P. falciparum* genome overall. Recombination breakpoints do, however, clearly associate with PQSs. We also show that variantly-expressed virulence gene families are associated with PQSs in several species of *Plasmodium* besides *P. falciparum*. Finally, we show that *Plasmodium* species possess an unusually limited set of putative G4 helicases.

## Results

### G-quadruplex-forming motifs in the *P. falciparum* genome are strongly associated with *var* genes

A search for PQSs in the genome of the reference strain of *P. falciparum*, 3D7, was previously published in 2009, finding 63 PQSs [17]. By searching the updated ‘version 3’ assembly of this genome [34], we found 80 PQSs (Additional file 1: Table S1), of which 35 were *var*-gene-associated, i.e. the PQS was either within a *var* coding sequence or the nearest predicted gene, within 2 kb of the PQS, was a *var* gene. 19 of these PQSs were inside a *var* coding sequence and 16 were within 2 kb of a *var* gene start site, with this latter group being exclusively in the upsB type of upstream region [17]. This represents a highly significant co-distribution of *var* genes and PQSs, compared to the expected distribution in a simulated genome in which *var* genes and PQSs occur at random (Table 1). PQSs were found throughout this work using the tool ‘QGRS Mapper (version 1)’ [35] with the same parameters as in the previous publication [17]: G_3_ N_(0–11)_ G_3_ N_(0–11)_ G_3_ N_(0–11)_ G_3_. The use of *N* ≤ 11 for the loops that separate guanine tracts represents a relatively relaxed algorithm because G4 formation becomes less favourable as the loops grow longer, and *N* ≤ 7 is frequently used when searching other genomes [36], although anything up to *N* = 25 has been used in some studies [37] (searching the *P. falciparum* genome with the more stringent *N* ≤ 7 criterion yielded only 31 PQSs (Additional file 1: Table S1)). Thus the most accurate predictive algorithm remains debatable, but Smargiasso et al. previously showed that ‘G_3_ N_(0–11)’ _motifs from the *P. falciparum* genome can fold into G4s under physiological conditions [17].

**Table 1:** Co-distribution of PQSs with variant-antigen-encoding gene families in *Plasmodium spp.*

*Plasmodium spp.*	No. genes in gene family	Mean distance from a PQS (kb)	Mean distance from a PQS in null dataset (kb)	Difference between actual & null data (kb)	Co-distribution (*p*-values)
*P. falciparum*	61	101.8	324.2	222.4	Y (*p* < 0.001)
*P. berghei*	217	356.3	414.2	57.9	Y (*p* = 0.021)

The distribution of distances between *var* or *pir* genes and their nearest PQS was compared to the distribution of distances in a simulated genome containing randomly-located genes. Differences between the actual and null datasets were assessed by Welch’s *t*-test (2-tailed)

### Highly stable G-quadruplex-forming motifs are not selected against in the *P. falciparum* genome

**Fig. 1:**
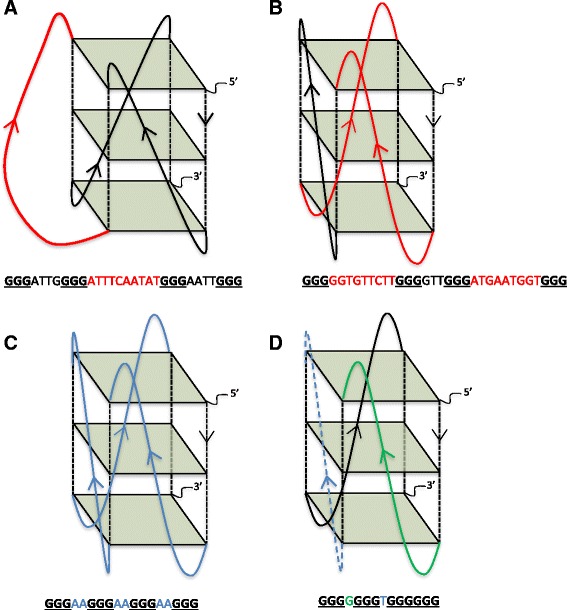
Schematic showing G-quadruplex DNA motifs that have different stabilities in model organisms. Examples of PQS sequences, with corresponding schematic structures, taken from the *P. falciparum* 3D7 genome (an example is shown of one of several structures that each sequence could adopt). Guanine tetrads are shown as green squares and guanine backbones as dashed black lines. Panels **a**-**d** demonstrate the four determinants that were shown to promote G4 stability and hence recombination events in vivo in *S. cerevisiae*: the location of the longest loop (*red*) being central (**a**) as opposed to lateral (**b**); a total loop length of 7 nucleotides or less (**c**); short loop lengths (*blue*) of only one or two nucleotides (**c**); and loops composed of a single pyrimidine (*blue, dashed*) as opposed to a single purine (*green, solid*) (**d**)

To assess whether *var*-gene-associated PQSs might have more recombinogenic properties than PQSs located elsewhere, we scored both groups for the criteria that were reported in the yeast model system to define particularly recombinogenic motifs [30]: loop lengths of only 1 or 2 nucleotides, total loop length ≤7 nucleotides, single pyrimidine rather than purine loops, and having the longest of the three loops in the central position rather than a flanking position (Fig. 1).

Contrary to the under-representation reported in other genomes, most of these features were not rare amongst PQSs in the 3D7 genome (Table 2). 15 out of 80 PQSs (19 %) had a total loop length of ≤7 nucleotides, which is close to the expected percentage (21 %) if total loop length was evenly distributed from 0 to 33 nucleotides. Similarly, 1- or 2-nucleotide loops comprised the expected proportions of the total, although only 6 out of 27 single-nucleotide loops were pyrimidines: less than half the expected number if pyrimidines and purines occurred in a 50:50 ratio. Finally, 32 out of 80 PQSs had their longest loop in the central position, again close to the expected one-third of the total. Therefore, there is little evidence that particularly stable and hence recombinogenic PQSs are selected against in the *P. falciparum* genome overall.

**Table 2:** Characteristics of putative G-quadruplex-forming sequences in the *P. falciparum* 3D7 genome

PQS group	Total # PQSs in group	# PQSs with total loop length ≤7	# of 1 or 2 nucleotide loops		# single pyrimidine (not purine) loops	# PQSs with a longest-central loop
			1 nt	2 nt		
*Var*-associated	35	2 (5.7 %)	4 (3.8 %)	4 (3.8 %)	4 (3.8 %)	23 (65.7 %)
Non-*var*-associated	45	13 (28.9 %)	23 (17.0 %)	18 (13.3 %)	2 (1.5 %)	9 (20 %)
Total	80	15 (18.8 %)	27 (11.3 %)	22 (9.2 %)	6 (2.5 %)	32 (40 %)
Signif. difference?		Y	Y		N	Y
*p*-value		0.0095	<0.0001		0.3959	<0.0001

*Var*-associated and non-*var*-associated groups of PQSs were scored for the four characteristics predicted to be G4-stabilizing: the table shows the number of PQSs in each group showing each characteristic. Differences between the scores for the two groups were assessed by Fisher’s exact test (2-tailed)

We then turned to the relative stabilities of *var*-associated versus non-associated PQSs. Among the 35 *var*-associated PQSs, 1- or 2-nucleotide loops and total loop lengths ≤7 were actually under-represented rather than over-represented. However, this group did contain the majority of the single pyrimidine loops and a significant majority of the longest-central loops. Since the conclusion varies depending on the characteristic examined, this does not constitute strong evidence that *var*-associated PQSs might be more recombinogenic than average. Notably, the power of all these comparisons is limited by small datasets (single pyrimidine loops, in particular, were too scarce for their skewed distribution to reach statistical significance (Table 2)).

To extend this analysis, we sought to investigate whether PQSs of any sort are under- or over-represented in the *P. falciparum* genome overall. Algorithms for predicting the PQS density in a genome are, however, not simple to establish: previous work has shown that randomly-generated genomes based simply on G/C and A/T content are poor models for real genomes because they do not account for variations in composition across the genome [36]. Indeed, although such ‘Bernoulli stream’ models have not previously been explored in genomes as strongly A/T-biased as *P. falciparum*, we found that a 23 Mb genome of 80.6 % A/T, representing *P. falciparum*, was predicted to contain no PQSs at all (Additional file 2: Figure S1). Modelling the sequence as windows of variable base composition, based on that of the real genome, was also inadequate because it still underestimated the real number of PQSs and furthermore the outcome was highly dependent on the size of window chosen (Additional file 2: Figure S1). Finally, we modelled the genome via a Markov chain that incorporated real base dyad frequencies (i.e. the real probability of any one base following another). This type of model has previously been shown to generate a number of PQSs similar to the number seen in a real human genome [36], yet still highly dependent on the size of the sliding window used. When replicated for the *P. falciparum* genome, results were consistent with those previously published: i.e. the number of PQSs decreased sharply as the window size increased from 50 to 4000 nucleotides (Additional file 2: Figure S1). Due to the subjective choice of window size, we were unable to use this model with confidence to determine whether the *P. falciparum* genome would be expected, ‘at random’, to contain more or less than 80 PQSs.

### Mitotic recombination events in the *P. falciparum* genome tend to occur close to G-quadruplex forming motifs

Since theoretical analysis yielded no strong conclusions about the representation or the recombinogenic potential of PQSs in *P. falciparum*, we then proceeded to analyze experimental data, collating all the published recombination breakpoints found to occur during asexual mitotic growth in *P. falciparum* (Additional file 3: Table S2). Two independent studies have used ‘clone tree’ approaches followed by whole-genome sequencing of the 3D7 reference strain to map such recombination events: this involves growing a culture of parasites over many months, repeatedly cloning the culture via limiting dilution and sequencing the progeny at each round of cloning, thus identifying changes in the genome that accumulated during each growth period prior to cloning. Bopp et al. [12] identified 4 interchromosomal translocations between subtelomeric *var* genes, 3 translocations between noncoding subtelomeric regions, and 19 intrachromosomal events (indels) where both breakpoints were identified on the same chromosome. In a larger study, Claessens et al. [11] identified 6 subtelomeric *var*-coding translocations, 12 subtelomeric noncoding translocations, 13 recombinations in a chromosome-internal tandem array of *var* genes on chromosome 4, and 18 indels with non-coding breakpoints. These authors also mapped *var*-coding recombination events in the progeny of the 3D7/HB3 genetic cross, finding an additional 12 events that had occurred mitotically in the 3D7 parent strain prior to the actual cross. Thus, a total of 87 events has been recorded (i.e. 173 breakpoints, one breakpoint being impossible to map in the current genome assembly). Of these, the majority lie in sub-telomeres and are associated with *var* genes – this may be because recombination is especially frequent in sub-telomeres, or possibly because DNA repair events in these regions are more likely to yield viable genomes.

**Fig. 2:**
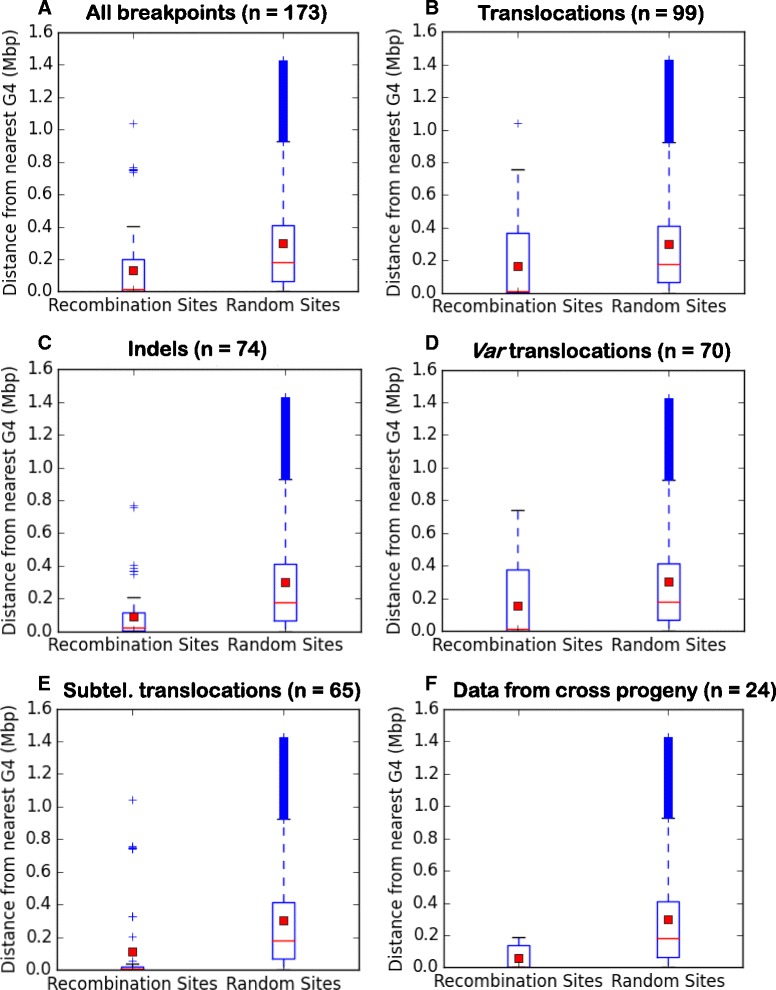
G-quadruplex DNA motifs associate with mitotic recombination breakpoints in the *P. falciparum* 3D7 genome. Box plots show the distribution of distances between recombination breakpoints and PQSs for the actual dataset (‘recombination sites’) and the null dataset (‘random sites’, *n* = 1 million, sampled equally across the genome). Red squares indicate means, red lines indicate medians, blue boxes indicate interquartile ranges and + indicate datapoints lying outside this range. **a** All breakpoints, *n* = 173, **b** All translocations, *n* = 99, **c** All indels, *n* = 74, **d** *Var*-associated translocations only, *n* = 70, **e** Sub-telomeric translocations only, *n* = 65, **f** *Var*-coding events detected in the progeny of the genetic cross only, *n* = 24

To quantify the association between recombination events and PQSs, we calculated the proximity of a million random genomic locations to their nearest PQS, and then compared this model with the distances of actual breakpoints to a PQS (Additional file 4: Table S3). Breakpoints were highly over-associated with PQSs when compared to the null dataset: the median distance from a PQS was only 16 kb versus 180 kb in the model; the mean was 133 kb versus 301 kb (Fig. 2a, Table 3). We then divided all 173 breakpoints into subsets – all translocations, all indels, only the *var*-associated translocations, only the sub-telomeric translocations, and only events detected in the progeny of the genetic cross - and repeated the analysis. Different subsets varied somewhat in their average proximity to a PQS, but all subsets remained significantly associated with PQSs when compared to a null dataset (Table 3 & Fig. 2b-f). Notably, the subset of data from the genetic cross, where *only var*-coding events were mapped, gave the closest association: a median distance of 3 kb and mean of 56 kb. We also considered the possibility that different chromosomes may differ in their inherent recombinogenicity (either because they contain more *var* genes, or because of other unknown chromatin features) and therefore we repeated the analysis with biased sampling of the null dataset according to how many breakpoints had actually been found on each chromosome. This reduced the magnitude of difference between the actual and null data, but a strong association remained (Table 3).

**Table 3:** Mean and median distances between PQSs and recombination breakpoints in the *P. falciparum* 3D7 genome

Breakpoint type	Number	Mean distance from PQS (kb)	Mean distance from PQS in null data (kb)	Median distance from PQS (kb)	Median distance from PQS in null data (kb)	PQS association (median outside C.I.)
All	173	132.7	301 (Equal)	16.4	180.4 (Equal)	Y
			193.8 (Unequal)	C.I. 11.1–24.3	130.5 (Unequal)	Y
All translocations	99	163.6	300.7 (Equal)	13.5	180.1 (Equal)	Y
			193.4 (Unequal)	C.I. 5.9–53.9	130.4 (Unequal)	Y
Subtelomeric translocations only	65	109.5	300.1 (Equal)	4.8	180.6 (Equal)	Y
			208.8 (Unequal)	C.I. 3.5–8.9	129.7 (Unequal)	Y
All *var* coding translocations	70	156.6	301.1 (Equal)	14.7	180.6 (Equal)	Y
			181.7 (Unequal)	C.I. 4.1–18.5	125.7 (Unequal)	Y
Indels	74	91.4	300.9 (Equal)	20	179.9 (Equal)	Y
			193.4 (Unequal)	C.I. 11.3–28.4	130.3 (Unequal)	Y
*Var*-coding events in progeny of 3D7/HB3 cross	24	56.2	300.1 (Equal)	2.9	180.7 (Equal)	Y
			141.1 (Unequal)	C.I. 2.5–11.3	98.8 (Unequal)	Y
Analysis restricted to subtelomeres	119	91.3	133.5 (Equal)	9.1	22.8 (Equal)	Y
			122.1 (Unequal)	C.I. 6.3–15.4	18.8 (Unequal)	Y

Mean and median PQS-to-breakpoint distances are shown for each actual dataset and for two simulated null datasets: breakpoints distributed equally across the genome (equal), or breakpoints distributed according to the number actually observed per chromosome (unequal). The significance of the difference between each actual and null dataset is assessed by calculating a 95 % confidence interval (C.I.) around the sample median: a significant association is noted if the median of the null dataset is outside this confidence interval

**Fig. 3:**
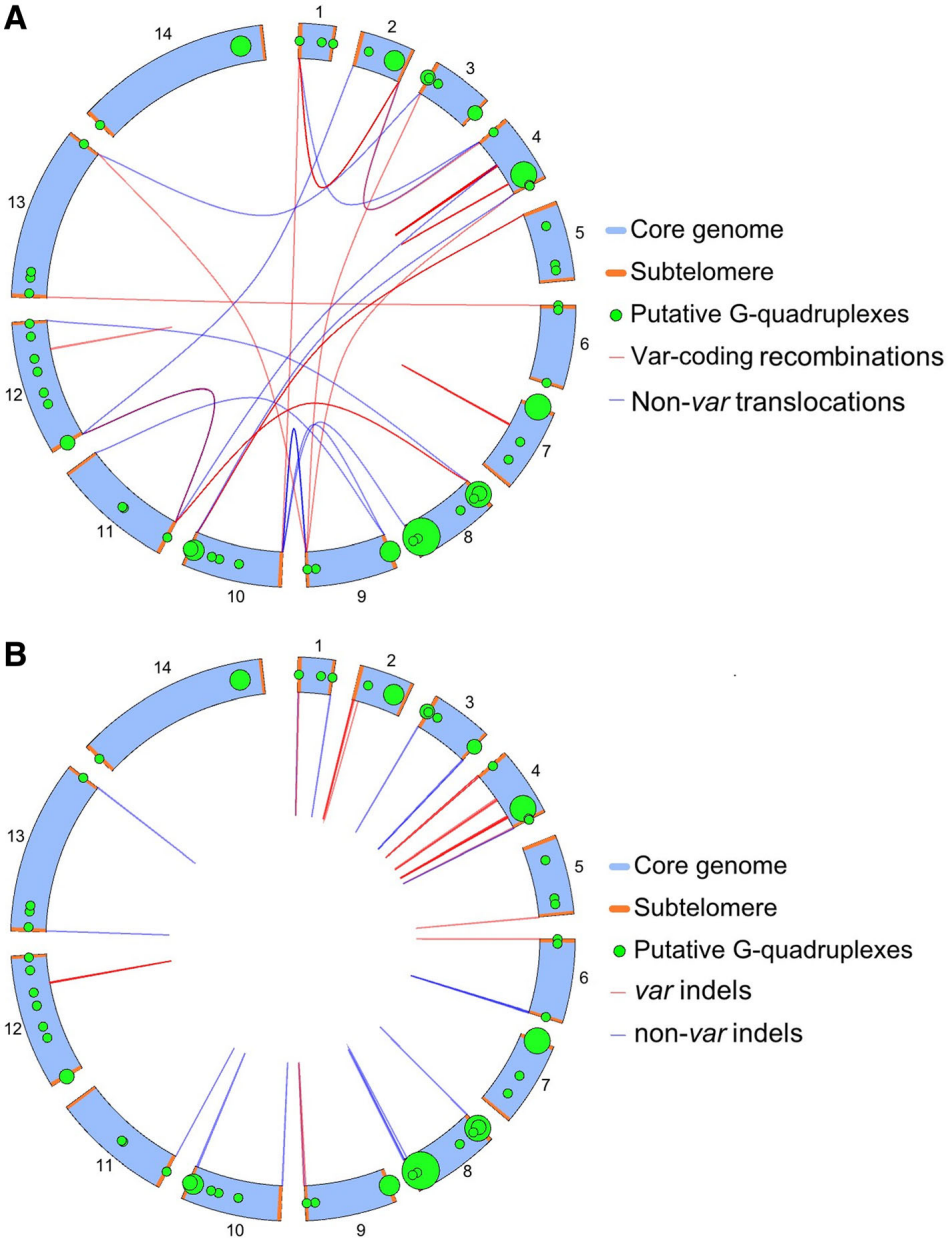
Schematics of PQSs and mitotic recombination events in the *P. falciparum* 3D7 genome. Schematics show the distribution of PQSs (*green circles*) across the 14 chromosomes, together with all recombination events recorded in the 3D7 genome (lines). **a** All *var* recombination events (*red*) and other non-*var* translocations (*blue*). **b** All indels, involving only a single chromosome: *var* (*red*) and non-*var* (*blue*). Circles representing PQSs are scaled in diameter according to the number found within each ~64 kb of the genome, represented by 1° of the 360° in this circular schematic. Overlapping circles occur in places where many PQSs lie within a single segment, making the circle large enough to overlap with adjacent segments

Finally, we noted that both recombination breakpoints and PQSs clearly cluster in subtelomeric regions, as seen in Fig. 3 and evidenced by the skewed distributions in Fig. 2. We therefore restricted our analysis to these subtelomeric regions, simulating a null dataset of a million breakpoints sampled exclusively within subtelomeres - i.e. within 65 kb of each chromosome end (this cutoff was defined to encompass all subtelomeric *var* genes because the most telomere-distal *var*, on chromosome 6, extends to 64.3 kb from the chromosome end). When this new null dataset was compared to the PQS proximities of the actual breakpoints occurring within subtelomeres, breakpoints were still over-associated with PQSs: median distance 9 vs. 23 kb and mean 91 vs. 133 kb (Table 3). Thus, the association transcended any a priori clustering of PQSs and breakpoints within subtelomeres. Furthermore, from the large difference between the means and medians it is clear that there are only a few breakpoints for which the nearest PQS lies a long way away on the chromosome: for the majority there is a proximal PQS within that same subtelomere.

### The association of G-quadruplex forming motifs with *var* genes is conserved across strains of *P. falciparum* and also in *P. reichenowi*

In addition to the reference strain 3D7, a second strain of *P. falciparum* has also been subjected to long-term growth and mapping of mitotic recombination events by Claessens et al. [11]. We repeated our analyses on this strain, Dd2, finding 61 PQSs in the Dd2 genome, of which 26 are *var*-gene-associated (Additional file 5: Table S4). This is somewhat fewer than the total of 80 PQSs found in 3D7 but the Dd2 strain has an unusually small *var* gene family (only 49 members were identified by Rask et al. in an analysis of several different *P. falciparum* strains, compared to 61 in 3D7 [38]). A similar proportion of the PQSs in Dd2 are *var*-gene-associated, with about half being gene-coding and half upstream, and the PQSs upstream of *var* genes are restricted to the upsB-type upstream regions, just as in 3D7. This therefore confirms that the clustering of PQSs in and around *var* genes is conserved across strains. Only a small number of recombination breakpoints were recorded in Dd2 compared to 3D7, and only *var*-coding breakpoints were mapped to the genome (Additional file 4: Table S3). There were 22 of these, of which we were able to map 19 to a chromosomal location (the annotation of the Dd2 genome being less complete than that of 3D7), plus an additional 30 breakpoints from the progeny of the Dd2/HB3 cross. Interestingly, the association between these breakpoints and PQSs was statistically significant if the null dataset was sampled equally across the genome, but it was not significant if the null dataset was sampled according to the number of breakpoints found on each chromosome, because the breakpoints found in Dd2 were very heavily biased towards a single chromosome (Additional file 6: Figure S2). This may be an artefact of the small size of this dataset, or it may be because recombination events in this strain really do tend to occur in a single focus: an intra-chromosomal *var* gene array on chromosome 8.

Since the PQSs in *P. falciparum* appear to be significantly clustered in and around *var* genes regardless of the strain examined, we investigated whether this phenomenon has been evolutionarily conserved by analyzing the genome of the closest sequenced relative, *P. reichenowi* (CDC strain). *P. falciparum* and *P. reichenowi* are very similar in genome size and A/T bias, and are largely syntenic, with *var* gene families that are highly diverse but analogous in their genomic arrangement and sequence features [31].

According to the G_3_ N_(0–11)_ algorithm, the *P. reichenowi* genome contains 53 PQSs (Additional file 7: Table S5), of which 33 are *var*-gene-associated. Thus, the total number of *var*-associated PQSs appears to be similar in both species; however, almost all of the *var*-associated PQSs in *P. reichenowi* are actually *var-*coding, whereas in *P. falciparum* about half of them are located upstream of upsB-type *var* genes: this group is entirely missing in *P. reichenowi* (Table 4). Incomplete genome assembly is unlikely to account for this, although the *P. reichenowi* genome does currently consist of more than 200 short contigs in addition to the 14 assembled chromosomes, and many *var* genes do remain on the contigs because highly homologous and repetitive sequences are difficult to assemble. These contigs contain many *var*-coding PQSs but they do not contain the ‘missing’ non-coding PQSs, despite the fact that upsB-type regions are present ([38], confirmed by BLAST search of *P. reichenowi* with upsB sequence from *P. falciparum,* data not shown). Notwithstanding the incomplete genome assembly, which prevented a full mathematical analysis of *var*/PQS association as per Table 1 for *P. falciparum*, it still seems clear that PQSs are heavily clustered with *var* genes in *P. reichenowi*: in fact 62 % of all PQSs are *var*-associated in *P. reichenowi*, compared to just 44 % in *P. falciparum* 3D7.

**Table 4:** Locations of PQS in the genomes of *P. falciparum*, *P. reichenowi* and *P. berghei*

		Genome	
	*P. falciparum* 3D7	*P. reichenowi* CDC	*P. berghei* ANKA
All PQS			
Within a gene	34	40	11
Upstream (within 2 kb)	37	5	30
Downstream (within 2 kb)	7	8	7
No gene within 2 kb	2	0	0
Total	80	53	48
*Var*-associated PQS			
Within a *var* gene	19	33 (inc. 1 pseudogene)	
Upstream (within 2 kb)	16	0	
Downstream (within 2 kb)	0	0	
Total *var* sequences	103^a^	103^a^	
*Var* genes	63^a^	64^a^	
Fragments/pseudogenes	6/34^a^	37/22^a^	
*Pir*-associated PQS			
Within a *pir* gene	3	0	0
Upstream (within 2 kb)	0	1	28
Downstream (within 2 kb)	0	1	6
Total *pir* sequences	226^a^	528^a^	299
*Pir* genes	189^a^	351^a^	217
Fragments/pseudogenes	0/37^a^	118/59^a^	0/82

Table shows numbers of PQS found in each *Plasmodium* genome analysed. ‘Fragments/pseudogenes’ refers to the numbers of incomplete, but recognizable, *var* gene sequences, which are designated as gene fragments or pseudogenes

^a^Indicates gene numbers taken from Otto et al. [31]

Concerning PQSs elsewhere in the core genome, there are fewer of these in *P. reichenowi* than in 3D7 *P. falciparum*, and this is very unlikely to be an artefact of genome assembly because the core genome is well-assembled. Certain genes are conserved across species complete with their associated PQSs: 5 such conserved loci were identifiable out of the 13 PQSs not associated with variant gene families. For example, the gene encoding circumsporozoite protein (a key surface antigen in the parasite’s sporozoite stage) has an upstream PQS in both species.

### Putative G-quadruplex forming motifs associate with the *sicavar* family in *P. knowlesi* and the *pir* family in *P. berghei*

The *var* gene family is restricted to the *Laverania* subgenus of *Plasmodium* [31] and is not present in most *Plasmodium* species, including the four other human malaria parasites *P. vivax*, *P. ovale*, *P. malariae* and *P. knowlesi*. Different variantly-expressed multigene families do exist in these species, although they are less well characterized in their form and function than the *var* genes. We sought to establish whether G4s might play a more general role in diversification and/or expression switching of multigene families by searching other *Plasmodium* genomes for G4s and assessing their association with non-*var* gene families.

The macaque parasite *P. knowlesi* - a zoonotic parasite of humans - has a unique virulence gene family called *sicavar* that encodes a variant surface antigen. *Sicavar* genes are unusual in that they are not primarily subtelomeric: they occur throughout the genome, co-distributed with abundant blocks of intrachromosomal telomere repeat sequence (ITSs), which are very rare in other species such as *P. falciparum* [39]. ITSs have inherent G4-forming potential because they consist of partially-degenerate GGGTT(C/T)A repeats. Accordingly, it has already been established that there is a strong association between *sicavar*s and PQSs: 86.5 % of the genes are within 10 kb of a PQS-rich ITS [39]. The *P. knowlesi* genome is also quite abundant in non-ITS PQSs, since its genome bias is very different from *P. falciparum* at 62.5 % A/T [39]. Indeed, the G_3_ N_(0–11)_ algorithm identifies a higher number of non-telomeric PQSs in just the smallest chromosome of *P. knowlesi* than in all 14 chromosomes of *P. falciparum* (93 versus 80 PQSs). Nevertheless, the PQS content of *P. knowlesi* is dominated by ITSs and the primary PQS/*sicavar* association appears here.

Besides the *var* and *sicavar* families, all sequenced *Plasmodium* species contain a more ‘ancient’ multigene family called *pir* [32, 33]. In *P. knowlesi* the *pir* genes are co-distributed with *sicavar*s and hence with ITSs [39], while in *P. falciparum* and *P. reichenowi* these genes – called *rifin*s and *stevor*s – are primarily subtelomeric and are co-distributed with *var* genes. Initial analysis suggested no specific association of *rifin*s and *stevor*s with PQSs, since no significant number of PQSs was found in *rifin*s and *stevors (*either coding regions or UTRs) (Table 4). For clarity, we turned to a *Plasmodium* species that has *pir* genes alone. Few *Plasmodium* genomes are sufficiently well assembled to permit this analysis but the genome of the rodent parasite *P. berghei* has recently been assembled to near-completeness [34]. This genome contains 48 PQSs (Additional file 8: Table S6), of which 34 (i.e. 71 %) are *pir*-associated (Table 4): a significant over-association of PQSs with *pir*s, as previously seen with *var*s (Table 1), although the larger size of the *pir* family weakens the association because many *pir*s are *not* PQS-associated. Notably, the distribution of PQSs in and around *pir* genes also differs from both *P. falciparum* and *P. reichenowi*: there are none within *pir* coding regions and the majority are closely upstream of *pir* genes (Table 4).

### *Plasmodium* species encode only a subset of the G-quadruplex helicases found in model organisms

DNA helicases in three different classes can unwind G4 motifs, allowing the passage of DNA and RNA polymerases [40]. *Plasmodium* genomes encode homologues of some, but not all, of these proteins. PIF1 is a highly efficient G4 helicase [41] which is widely conserved in eukaryotes from yeast to human, and indeed in prokaryotes [42], but no PIF1 could be detected via homology searching in any *Plasmodium* genome.

*Plasmodium* has two representatives of the ‘canonical’ RecQ class of G4 DNA helicases, which have been designated *Pf*BLM and *Pf*WRN in previously-published *in silico* characterization (Additional file 9: Figure S3, [17, 43–45]). RecQs are 3’–5’ helicases homologous to the bacterial RecQ, with five representatives found in humans: BLM, WRN, RECQ1, RECQ4 and RECQ5 [46]. Both *Plasmodium* homologues encode the ATP-binding and Helicase C-terminal (RCQ) regions that are characteristic of most RecQ helicases, but they lack a clear Helicase & RNaseD C-terminal (HRDC) region. This is found in only some members of the RecQ family and is involved in structure-specific DNA binding and protein-protein interactions [46]. It remains to be established, in the absence of this domain, whether these helicases can still bind to and unwind G4 motifs in *Plasmodium*.

**Fig. 4:**
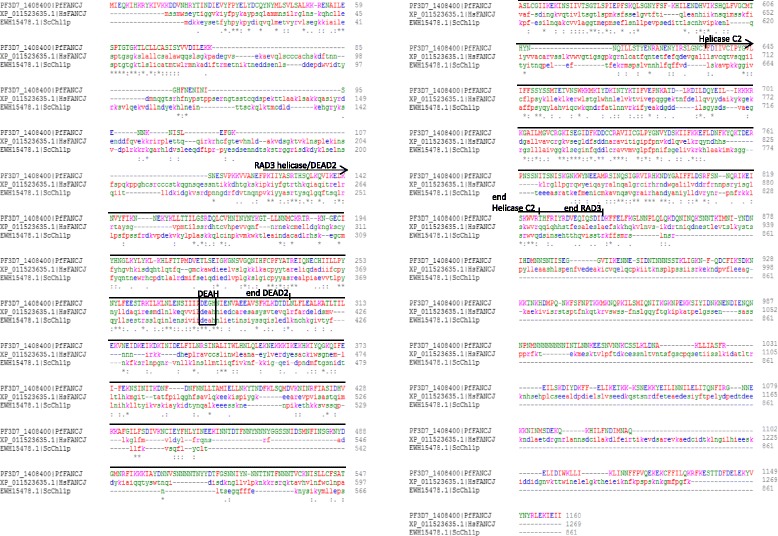
Alignment of the FANCJ helicase sequence in *Plasmodium spp. Pf*FANCJ is aligned with *H. sapiens* FANCJ and *S. cerevisiae* Chl1. Key protein domains and strictly conserved (*) or conserved (: or.) residues are marked. Amino acids are colour-coded by chemical properties: blue: acidic; magenta: basic; red: small, hydrophobic; green: hydroxyl,sulfhydryl,amine,G

Finally, the RAD3-family helicase FANCJ also has a putative homologue in *Plasmodium* genomes (Fig. 4). This 5’–3’ helicase resolves interstrand crosslinks as part of the Fanconi Anaemia pathway [47, 48], but it is now reported to have an independent role in metabolizing G4s as well [49]. *Plasmodium* genomes encode only one clear FANCJ homologue, although the human genome encodes a second helicase of the same family, RTEL1. *Pf*FANCJ shows 25 % identity with human FANCJ and moderate conservation throughout the central RAD3 helicase domain (DEAD2 and HelicaseC2 regions), but no conservation in the C-terminal region, which mediates interaction of human FANCJ with the breast cancer susceptibility protein BRCA1. The same region is absent in the *S. cerevisiae* homologue Chl1 and *Plasmodium*, like *S. cerevisiae,* encodes no clear homologue of BRCA1. *PfFANCJ* expression levels peak in the DNA-replicating trophozoite stages, as also seen for *PfBLM*, while *PfWRN* peaks in trophozoites and also schizonts [34]: the expected expression patterns for genes involved in DNA replication and repair.

## Discussion

This study explored the number and nature of PQSs found in malaria parasite genomes. Firstly, we found that the PQSs in the 3D7 strain of *P. falciparum* do not comply with a trend previously proposed from other genomes, for selection against highly stable and hence recombinogenic G4 motifs [30]. This also held true in the related species *P. reichenowi* (data not shown). Several possibilities may explain this: firstly, PQSs may simply be so rare in these highly A/T-rich genomes that they are not a problem for genome stability, or can be adequately unwound by G4 helicases. Secondly, *Plasmodium spp.* may be unusually tolerant of genome instability: the karyotypes of circulating field strains vary markedly [50] and heavily rearranged genomes can still sustain viable parasites in in vitro culture [51]. Thirdly, peculiarities of DNA metabolism or replication in *Plasmodium spp*. may mean that G4s with a high thermal stability are not actually highly recombinogenic. This latter seems unlikely because repair of DNA breaks via homologous recombination is heavily favoured in *Plasmodium*: classical non-homologous end-joining is actually absent, although a microhomology-mediated end-joining pathway does exist [52]. Nevertheless, little is known about the dynamics of DNA polymerases in *Plasmodium* and if these differ from better-characterized model systems, the problem of replicating through G4s could be reduced.

We next sought empirical evidence for any role of G4s in recombination and generation of diversity in *Plasmodium* genomes, by assessing whether recombination breakpoints tend to occur close to PQSs. We found that breakpoints are indeed highly associated with PQSs. Furthermore, subtelomeric translocations and indels were most closely associated: it may be that G4-induced breaks have a particular tendency to be repaired using nearby DNA and that nuclear architecture is therefore influential. The clustering of subtelomeres and the existence of chromosomal ‘territories’ within the nucleus [53] would favour subtelomeric recombination between different chromosomes [14], and recombination events within the same chromosome, respectively.

PQSs and *var* genes are closely co-distributed in both *P. falciparum* and *P. reichenowi* genomes, with both features being clustered in sub-telomeres. Clustering of PQSs in subtelomeric regions is not generally reported in other genomes, suggesting that this co-distribution might be positively selected in *P. falciparum* specifically because it enhances *var* gene recombination. It is also notable that the PQSs in all *var* upsB regions are highly conserved [17], and that they lie in one of the most conserved areas of the upsB sequence, further suggesting a positively-selected functional role. Nevertheless, a co-distribution could still occur coincidentally, for other evolutionary reasons, so we investigated whether the PQS/breakpoint association ‘transcended’ the underlying co-distribution by restricting our analysis to subtelomeric regions and found that it did – further supporting our hypothesis. A previous analysis of *var* gene recombination, using a set of recombination events found in genetic-cross progeny which largely overlaps with the dataset analysed here, found that recombination occurred preferentially near to imperfect palindrome sequences that tend to form DNA hairpins [13]. These are much more abundant than PQSs, but they tend to be found at *var* genes (specifically at domain boundaries within *var* genes), and they can stall replication and thus promote recombination – albeit perhaps as a common and mild, rather than rare and severe, block to replication forks. Different DNA processing proteins may be required to resolve these two events (e.g. G4-specific helicases in the case of G4s) but they may ultimately stimulate the same recombination pathways if a fork breaks down and a DNA break does occur.

Mechanistic questions remain about exactly how a G4-induced DNA break would be repaired, since DNA repair is not well characterized in *Plasmodium*. By analogy with model systems, there could be gene conversion using another *var* gene as a template; break-induced replication converting an entire chromosome end to the sequence from another chromosome; or single-strand annealing with break resection until a homologous sequence is found at which to reanneal, resulting in deletion of the intervening sequence. There is direct evidence in *P. falciparum* for at least the first two options [11] and resection of at least 50 kb has been observed during break repair in *S. cerevisiae* [54]. In fact, long tracts of resection or branch migration may be heavily favoured in *P. falciparum* in order to find the ~80 bp of homology required for two *var* genes to recombine [11], and this could explain why many recombination breakpoints are found a few kb away from a PQS rather than exactly at the PQS site. Genome-wide, the mean distance between a breakpoint and its nearest G4 was ~133 kb, but with a strong skew towards shorter distances so the median distance was only ~16 kb and 63 % of all breakpoints occurred less than 50 kb from a PQS. It is therefore mechanistically possible that G4s could be responsible for a large proportion of observed recombination events. (Importantly, we analyzed all translocations and indels as two individual breakpoint events, because the end result does not reveal which of the two ends represented the ‘initiating’ break event.)

Having established that G4 motifs may indeed be a driver of recombination events in the *P. falciparum* 3D7 genome, we investigated whether they might play a similar role in other *Plasmodium* genomes. First we examined the only other *P. falciparum* strain in which breakpoints have been experimentally mapped, Dd2, and found a similar association, although this dataset was influenced by the strong clustering of recombination events on a single chromosome – which may reflect a genuine bias of recombination dynamics in the Dd2 strain, or an artefact of limited data.

Experimental data of this type do not exist for other *Plasmodium* species, but we nevertheless examined other genome sequences for co-distribution of PQSs with multigene families. If antigen-encoding gene families other than *var* can all benefit from hyper-recombination to increase their diversity, and if G4 motifs can drive this recombination, then PQSs would be expected to associate with antigen-encoding gene families across *Plasmodium* genomes. This was indeed true in *P. reichenowi* (*var* genes), *P. knowlesi* (*sicavar* genes), and *P. berghei* (*pir* genes). The association transcends the possibility discussed above of ‘*a priori*’ clustering of PQSs at chromosome ends because the *sicavar* family, which is not subtelomeric, is still co-distributed with PQSs. Some intriguing differences in the details of PQS distribution emerged from the analysis of non-*falciparum* genomes: *P. reichenowi* has many more *var*-coding PQSs than *P. falciparum*, but lacks any PQSs in *var* 5’UTRs. *P. berghei*, by contrast, has no PQSs within *pir* coding genes, but many in *pir* UTRs. Differences in the evolutionary pressures for diversification of different gene families, or differences in the exact mechanism of that diversification, may be at play here.

Turning again to repair mechanisms, it has been shown in model systems that some G4s must be processed by structure-specific helicases to prevent polymerase stalling and potential replication fork collapse. In the absence of such helicases, G4-induced instability is greatly exacerbated in *S. cerevisiae* and indeed in human disease syndromes [55]. Therefore, if G4 helicases vary in their expression or activity in *Plasmodium spp*., this could alter recombination rates amongst antigen-encoding genes, affecting important clinical phenotypes like parasite virulence and the ability to maintain chronic infections.

We examined *Plasmodium* genomes for potential G4-processing helicases and found that *Plasmodium* lacks a recognizable homologue of the highly efficient G4 helicase PIF1. This gene is broadly conserved across eukaryotes and it seems unlikely that it was lost simply because it is redundant in G4-poor genomes, since not all *Plasmodium* genomes are G/C poor. *Plasmodium spp.* do, however, have two conserved homologues for RecQ helicases, designated *Pf*BLM and *Pf*WRN, although neither is yet established as a functional homologue of a particular enzyme from the RecQ family in mammals [43]. Molecular-genetic work (underway in our group) is needed to establish their exact roles – e.g. replication-coupled G4 processing, transcription-coupled or ‘surveillance’ G4 processing, telomere maintenance, etc. The single FANCJ helicase homologue in *Plasmodium* also remains uncharacterized, but by analogy with model systems it may play a replication-coupled G4-processing role [40].

## Conclusion

This is the first study to thoroughly examine the distribution of putative G-quadruplex-forming sequences in multiple *Plasmodium* genomes. The findings support the concept that G4s are involved in recombination and diversification of antigen-encoding gene families in this important protozoan pathogen.

## Methods

### Statistical analysis of the co-distribution of PQSs and multigene (*var* or *pir*) families

To determine whether there is a statistically significant association between the locations of *var*/*pir* genes and PQSs, the distance of every gene from its nearest PQS was calculated, measuring from the start or end of the PQS and scoring ‘0’ for genes with a PQS within their coding sequence. A set of genes with random positions was then simulated as a comparator to test the null hypothesis of no association. The simulated *var*/*pir* genes were generated with sizes normally distributed around the mean size of the actual gene families (since gene sizes within these families do appear normally distributed). Datasets of a million data points were built by calculating the distance of a million simulated genes from a PQS. The actual dataset and the simulated datasets were then analyzed to determine the mean distance from PQSs, with the actual mean distance being compared to the null situation. The statistical significance of each difference was assessed using Welch’s *t*-test (2-tailed), with significance set at *p* ≤ 0.05.

In the case of *P. berghei*, in which 35 of the 217 *pir* genes are assigned to five super-contigs in the current genome assembly, these contigs were treated as discrete chromosomes. There were also four *P. berghei* chromosomes that had no PQSs, so *pir*s on these chromosomes had to be excluded because the ‘distance to nearest PQS’ did not exist.

### Statistical analysis of the characteristics of var-associated versus non-associated PQSs

PQS characteristics were scored as follows, aiming to award higher numbers to PQSs with highly-stabilizing features. A) loop lengths of only 1 or 2 nucleotides: score ‘1’ for each 2 nt loop and ‘2’ for each 1 nt loop. B) total loop length ≤7 nucleotides: score ‘1’ if yes, ‘0’ if no. C) single pyrimidine rather than purine loops: score ‘1’ for each single-pyrimidine loop, ‘0’ for single purines. D) longest loop in the central position rather than a flanking position: score ‘1’ if yes, ‘0’ if no. These sets of scores for the *var*-associated and non-associated groups of PQSs were then assessed for significant differences using Welch’s *t*-test (2-tailed), with significance set at *p* ≤ 0.05.

### Modelling to predict numbers of PQS in Plasmodium genomes

In order to predict the likely number of PQSs in a given DNA sequence, we followed the approach used by Huppert et al. to model the human genome [36], applying it to the *P. falciparum* genome. The general methodology is to generate simulated genomes with similar statistical properties to the *P. falciparum* 3D7 genome; the number of PQSs within these simulated sequences is then calculated using a quad-parsing algorithm.

A naïve approach to generating genomes with a similar content to *P. falciparum* is simply to count the number of occurrences of each base in the reference genome (80.6 % A/T in this case) and use this frequency data to probabilistically produce a sequence of length N base-by-base, a so-called Bernoulli stream. Since this process aggregates across the whole genome, local variance in base density is lost. However, this problem can be mitigated in two ways. First, as noted in [36], the varying A/T density can be incorporated into the model by using a windowed Markov chain. We also considered that the Markov assumption may be unnecessary and that windowing the Bernoulli scheme may also help to maintain the heterogeneity of A/T distribution in the model.

The naive Bernoulli scheme generator creates N-base sequences based on an input of A/T density as a percentage of the whole genome. We analyzed the output of this generator for a range of A/T densities, from 0 % to 90 % in 10 % increments, counting the number of PQSs found in the data using a simple regular expression (regex) quad-parser.

The windowed Bernoulli stream generator analyses the reference genome and counts base frequencies in blocks of *k* bases, where *k* is the window size and *k* ∈ {50, 75, 100, 150, 200, 400, 1000, 2000, 4000}. We then generated 10 random simulated genomes of 22.9 Mb length for each window size and counted the number of PQSs present in each sequence.

The Markovian generator analyses the reference genome and counts base pairs in blocks of *k* bases, where *k* is the window size and *k* ∈ {50, 75, 100, 150, 200, 400, 1000, 2000, 4000}. For each window, the base dyad frequencies are collected (the likelihood that base B follows base A in the window) and thus a statistical model of the whole genome under the Markov assumption is constructed. For each window size we then generated 10 random genomes, equivalent in length to 3D7, using the likelihoods for each base in each window to generate the next item in the sequence. Finally, the number of PQSs present in the simulated sequences was counted.

All modelling, parsing and statistical analysis was performed using Python 2.7.10. Code is available at https://github.com/StanDeSiecle/breakpoint-PQS-distances. For each generator scheme, we used the same quad-parser regex, GGG.{1,11}?GGG.{1,11}?GGG.{1,11}?GGG.

### Collation of data on recombination breakpoints

3D7: All previously-published experimentally-determined breakpoints [11, 12] were originally mapped in version 2 of the *P. falciparum* 3D7 genome assembly. We re-mapped all of these breakpoint co-ordinates to version 3 of the *P. falciparum* 3D7 genome assembly, since the PQS dataset was determined in version 3. For each indel two recombination events were defined; one at the start and one at the end of the genomic region spanned by the indel. Likewise, each translocation was defined by two breakpoint events on two separate chromosomes. Each of the two breakpoints involved in a translocation or indel was defined as *var*-associated if one or both of the breakpoints was located either within a *var*-coding region or if the nearest gene was a *var* and was within 2 kb of the breakpoint. Similarly, each of the two breakpoints involved in a recombination event was defined as subtelomeric if one or both of the breakpoints occurred within 65 kb of a chromosome end. Not all translocations had been mapped to single-base-pair resolution by Claessens et al. [11]: where only the start and end co-ordinate of an identity block was listed, this whole region was taken as the breakpoint. Recombination events found in the progeny of the 3D7/HB3 genetic cross were also included if both breakpoint events occurred in the 3D7 genome (and were therefore present in the 3D7 parent prior to the genetic cross).

Dd2: V*ar* exon 1 breakpoints identified by Claessens et al. [11] were re-mapped in the most complete assembly of Dd2 available (ftp://ftp.sanger.ac.uk/pub/pathogens/Plasmodium/falciparum/PF3K/PilotReferenceGenomes/DraftAnnotation/PfDd2/ (Accessed Apr 2016)). Again, breakpoints in the Dd2 genome that were mapped in progeny of the Dd2/HB3 genetic cross were also included, since they occurred mitotically within the parent strain prior to the cross.

### Statistical analysis of correlation between PQSs and recombination breakpoints

To determine whether there is a statistically significant association between recombination breakpoints and PQSs, the distance of every breakpoint from its nearest PQS was calculated, measuring from the start or end of the PQS. Three set of sites with random positions were then simulated as comparators in order to test the null hypothesis of no association. Datasets of a million data points were built by randomly sampling a million loci from the 3D7 genome in three ways: i) equally across the genome, in a distribution based on the lengths of the 14 chromosomes; ii) unequally, based on the observed distribution of recombination sites on each chromosome (this resulted, for example, in a heavier sampling of chromosome 4, which is one of the smaller chromosomes but which has many recombination breakpoints in its intrachromosomal *var* gene array); iii) within the terminal 65 kb of all chromosomes to yield a subtelomeric-restricted null dataset. In all null datasets, the distance of each locus from a PQS was again calculated.

The actual and simulated datasets were then analyzed to determine the mean and median distance from PQSs, with the actual values being compared to the null situation. For the comparison with the subtelomeric null dataset (iii), only actual breakpoints that occurred within 65 kb of each chromosome end were included. Significance was tested by calculating a 95 % confidence interval around the median and determining whether the null median fell outside this interval. For means, the strong non-normal skew of all datasets invalidated the *t*-test as a way of testing differences between null and sample means and therefore these tests are not presented (although they did give highly significant results for most datasets).

### Homology searches and alignments

To identify homologues of potential G4 helicases, BLAST searches were carried out on the database of all sequenced *Plasmodium* genomes, PlasmoDB [34], using the following protein sequences: *S. cerevisiae* Pif1 and Rrm3 and *H. sapiens* PIF1; *S. cerevisiae* Sgs1 and *H. sapiens* BLM and WRN; *H. sapiens* FANCJ. Alignments were made using Clustal Omega (www.ebi.ac.uk/Tools/msa/clustalo/).

## Additional files

Additional file 1: Table S1. Putative G-quadruplex-forming sequences in the *P. falciparum* 3D7 (version 3) genome. Genome searched using ‘QGRS Mapper’ [35] with the parameters G_3_ N_(0–11)_ G_3_ N_(0–11)_ G_3_ N_(0–11)_ G_3_ [17]. RC, reverse complement. Orange-highlighted rows denote PQSs that also meet the more stringent G_3_ *N* ≤ 7 criterion. (XLSX 21 kb)
Additional file 2: Figure S1. Modelling of the number of PQSs expected in the *P. falciparum* genome. A) Expected numbers of PQSs in 23 Mb genomes with a range A/T biases, modelled from a Bernoulli stream of A/T/G/C as described by Huppert et al. [36]. B) Outputs from a windowed Benoulli stream generator and from a Markov model based on actual base dyad frequencies in the *P. falciparum* genome. These models yield a number of PQSs that is highly dependent on the size of the sliding window analysed. Ten repeats of the analyses are plotted for each window size from 50 to 4000 bp. An analogous model, applied to the human genome, yielded the ‘real’ number of PQSs when the window size was ~150-200 bp [36]. These models do not attempt to exclude the several-hundred telomeric PQSs that occur in the *P. falciparum* genome, in addition to the 80 non-telomeric PQSs, and the ‘real’ number in the *P. falciparum* genome is therefore is found by the Markov model at a similar window size to the size which gave maximum accuracy in the human genome. (PDF 214 kb)
Additional file 3: Table S2. Recombination breakpoints occurring in *P. falciparum* 3D7 and Dd2 strains during long-term culture. Experimentally determined breakpoints were collated from Bopp et al. and Claessens et al. [11, 12]. For each breakpoint the following is listed: genomic location in the 3D7 v3 genome assembly (chromosome number and breakpoint start and end co-ordinates on that chromosome), the length of the breakpoint if not mapped to single-base-pair resolution, the type of breakpoint (translocation or indel), whether the recombination event that breakpoint was involved in is *var*-associated, whether the recombination event that breakpoint was involved in is subtelomeric, which publication the breakpoint data was taken from and which recombination event that breakpoint belongs to. (XLSX 21 kb)
Additional file 4: Table S3. Distances between recombination breakpoints in *P. falciparum* 3D7 and their nearest PQS. Recombination breakpoints and PQSs shown here are detailed in Additional file 1: Table S1 and Additional file 2: Table S2 respectively. (XLSX 16 kb)
Additional file 5: Table S4. Putative G-quadruplex-forming sequences in the *P. falciparum* Dd2 genome. Genome searched using ‘QGRS Mapper’ [35] with the parameters G_3_ N_(0–11)_ G_3_ N_(0–11)_ G_3_ N_(0–11)_ G_3_ [17]. (XLSX 18 kb)
Additional file 6: Figure S2. Analysis and boxplots showing the association between PQSs and mitotic recombination breakpoints in the *P. falciparum* Dd2 genome. A) Mean and median PQS-to-breakpoint distances are shown for each actual dataset and for two simulated null datasets: breakpoints distributed equally across the genome (equal), or breakpoints distributed according to the number actually observed per chromosome (unequal). The significance of the difference between each actual and null dataset is assessed by calculating a 95 % confidence interval around the sample median: a significant association is noted if the median of the null dataset is outside this confidence interval. B) Box plots show the distribution of distances between recombination breakpoints and PQSs for the actual dataset (‘recombination sites’) and the null dataset (‘random sites’, *n* = 1 million, sampled equally across the genome). Red squares indicate means, red lines indicate medians and blue boxes indicate interquartile ranges. C) Box plots as in (B), using a null dataset, sampled according to the number of breakpoints actually found on each chromosome. (PDF 210 kb)
Additional file 7: Table S5. Putative G-quadruplex-forming sequences in the *P. reichenowi* CDC genome. Genome searched using ‘QGRS Mapper’ [35] with the parameters G_3_ N_(0–11)_ G_3_ N_(0–11)_ G_3_ N_(0–11)_ G_3_ [17]. (XLSX 16 kb)
Additional file 8: Table S6. Putative G-quadruplex-forming sequences in the *P. berghei* ANKA genome (version 3). Genome searched using ‘QGRS Mapper’ [35] with the parameters G_3_ N_(0–11)_ G_3_ N_(0–11)_ G_3_ N_(0–11)_ G_3_ [17]. (XLSX 15 kb)
Additional file 9: Figure S3. Alignment of RecQ helicase sequences in *Plasmodium spp. Pf*BLM and *Pf*WRN are aligned with *H. sapiens* BLM and WRN, *S. cerevisiae* Sgs1 and *E.coli* RecQ. The seven conserved motifs found in all superfamily 1 & 2 helicases are boxed, and strictly conserved (*) or conserved (: or.) residues are marked. For more detailed discussion of the sequence features in these genes, see prior publications [43, 44]. (PDF 260 kb)

## Abbreviations

G4: G-quadruplex
HRDC: Helicase & RNaseD C-terminal
ITS: Intrachromosomal telomere repeat sequence
kb/Mb: Kilo/megabases
PfEMP1: *Plasmodium falciparum* Erythrocyte Membrane Protein 1
pir: *Plasmodium* interspersed repeat
PQS: Putative quadruplex sequence
RCQ: Helicase C-terminal
