# Supplementary figures and images for: Recombination events among virulence genes in malaria parasites are associated with G-quadruplex-forming DNA motifs
Source: BMC Genomics. 2016 Nov 3;17:859. doi: 10.1186/s12864-016-3183-3 (PMC5093961; doi:10.1186/s12864-016-3183-3)

**A**

| %A/T | Number of PQS | PQS per Mb |
|------|---------------|------------|
| 0    | 175223        | 7618       |
| 10   | 91369         | 3973       |
| 20   | 39176         | 1703       |
| 30   | 13051         | 567        |
| 40   | 3252          | 141        |
| 50   | 583           | 25         |
| 60   | 42            | 2          |
| 70   | 1             | 0          |
| 80   | 0             | 0          |
| 90   | 0             | 0          |

**B**

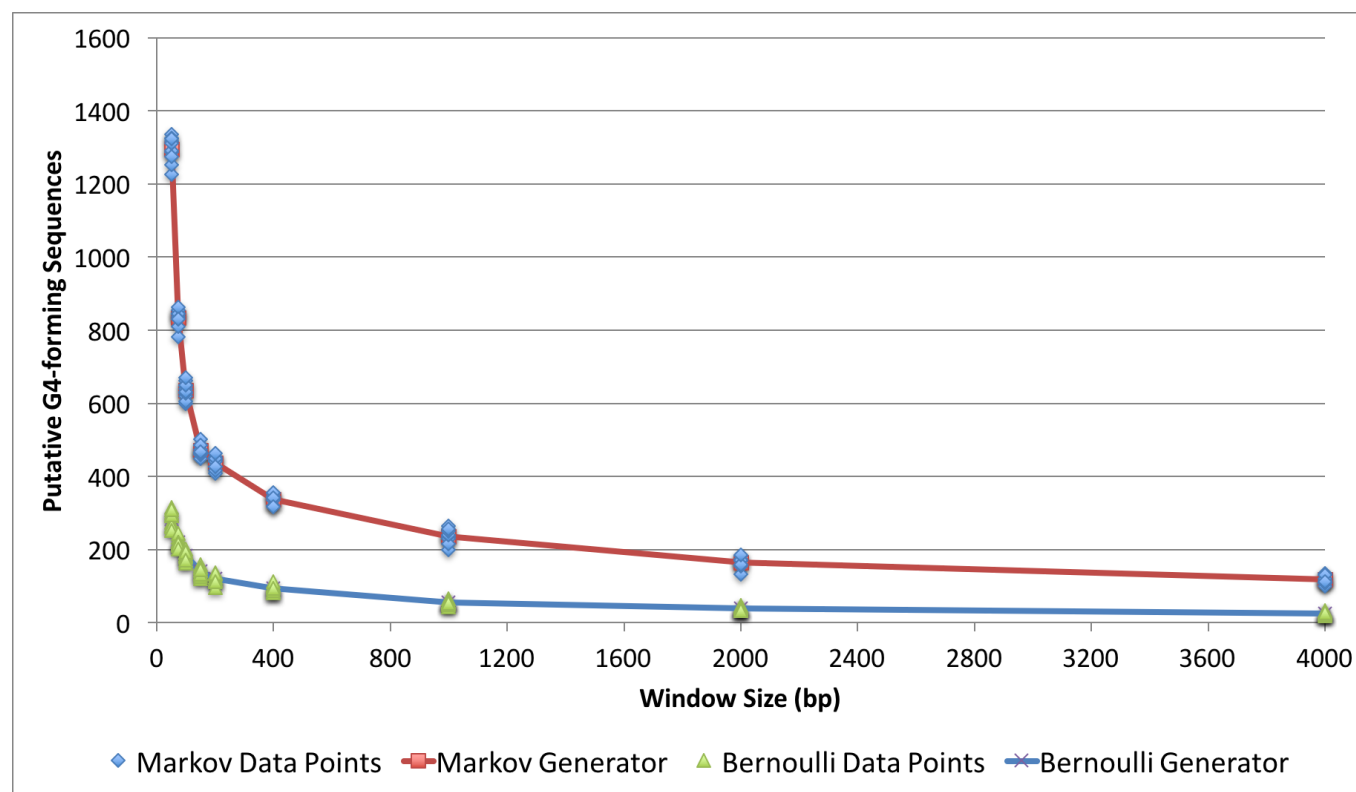

Supplement: Additional file 2: Figure S1. — Modelling of the number of PQSs expected in the P. falciparum genome. A) Expected numbers of PQSs in 23 Mb genomes with a range A/T biases, modelled from a Bernoulli stream of A/T/G/C as described by Huppert et al. [36]. B) Outputs from a windowed Benoulli stream generator and from a Markov model based on actual base dyad frequencies in the P. falciparum genome. These models yield a number of PQSs that is highly dependent on the size of the sliding window analysed. Ten repeats of the analyses are plotted for each window size from 50 to 4000 bp. An analogous model, applied to the human genome, yielded the ‘real’ number of PQSs when the window size was ~150-200 bp [36]. These models do not attempt to exclude the several-hundred telomeric PQSs that occur in the P. falciparum genome, in addition to the 80 non-telomeric PQSs, and the ‘real’ number in the P. falciparum genome is therefore is found by the Markov model at a similar window size to the size which gave maximum accuracy in the human genome. (PDF 214 kb) [file 12864_2016_3183_MOESM2_ESM.pdf]
