# Supplementary material for: Recombination events among virulence genes in malaria parasites are associated with G-quadruplex-forming DNA motifs
Source: BMC Genomics. 2016 Nov 3;17:859. doi: 10.1186/s12864-016-3183-3 (PMC5093961; doi:10.1186/s12864-016-3183-3)

A

Breakpoint type	Number	Mean distance from PQS (kb)	Mean distance from PQS in null data (kb)	Median distance from PQS (kb)	Median distance from PQS in null data (kb)	PQS association
All	49	306.9	590.4(Equal)	291.5	304.6 (Equal)	Y
			303.8 (Unequal)	C.I. 287.7-302.9	223.1 (Unequal)	N

B

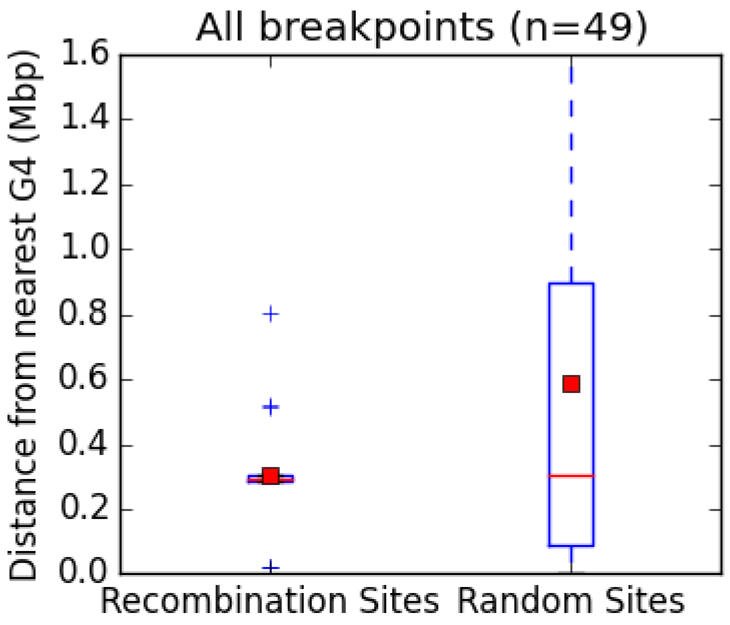

C

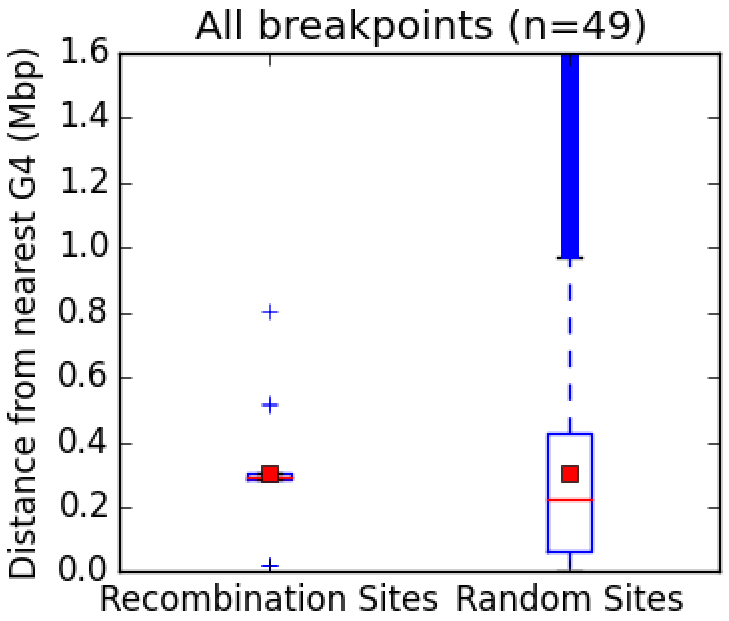

Supplement: Additional file 6: Figure S2. — Analysis and boxplots showing the association between PQSs and mitotic recombination breakpoints in the P. falciparum Dd2 genome. A) Mean and median PQS-to-breakpoint distances are shown for each actual dataset and for two simulated null datasets: breakpoints distributed equally across the genome (equal), or breakpoints distributed according to the number actually observed per chromosome (unequal). The significance of the difference between each actual and null dataset is assessed by calculating a 95 % confidence interval around the sample median: a significant association is noted if the median of the null dataset is outside this confidence interval. B) Box plots show the distribution of distances between recombination breakpoints and PQSs for the actual dataset (‘recombination sites’) and the null dataset (‘random sites’, n = 1 million, sampled equally across the genome). Red squares indicate means, red lines indicate medians and blue boxes indicate interquartile ranges. C) Box plots as in (B), using a null dataset, sampled according to the number of breakpoints actually found on each chromosome. (PDF 210 kb) [file 12864_2016_3183_MOESM6_ESM.pdf]
