# Supplementary material for: Recombination events among virulence genes in malaria parasites are associated with G-quadruplex-forming DNA motifs
Source: BMC Genomics. 2016 Nov 3;17:859. doi: 10.1186/s12864-016-3183-3 (PMC5093961; doi:10.1186/s12864-016-3183-3)
Supplement: Additional file 9: Figure S3. — Alignment of RecQ helicase sequences in Plasmodium spp. PfBLM and PfWRN are aligned with H. sapiens BLM and WRN, S. cerevisiae Sgs1 and E.coli RecQ. The seven conserved motifs found in all superfamily 1 & 2 helicases are boxed, and strictly conserved (*) or conserved (: or.) residues are marked. For more detailed discussion of the sequence features in these genes, see prior publications [43, 44]. (PDF 260 kb) [file 12864_2016_3183_MOESM9_ESM.pdf]

```
PF3D7_0918600|PfBLM     ------------------------------------------------------------ 0
PF3D7_1429900|PfWRN     ------------------------------------------------------------ 0
NP_001274175.1|HsBLM    maavpqnnlqeqlerhsartlnnklslskpkfsgftfkkktssdnnvsvtnvsvaktpvl 60
NP_000544.2|HsWRN       ------------------------------------------------------------ 0
DAA10088|ScSgs1         mvtkpshnlrrehkw-----lket------------------------------------ 19
AAA24517|EcRecQ         ------------------------------------------------------------ 0

PF3D7_0918600|PfBLM     ------------------------------------------------------------ 0
PF3D7_1429900|PfWRN     ------------------------------------------------------------ 0
NP_001274175.1|HsBLM    rnkdvnvtedfsfseplpnttnqqrvkdffknapagqetqrggsksllpdflqtpkevvc 120
NP_000544.2|HsWRN       ------------------------------------------------------------ 0
DAA10088|ScSgs1         --atlqedkdfvfqaiqkhiankrpktnsppttpskdecgpg-ttnfitsipa------- 69
AAA24517|EcRecQ         ------------------------------------------------------------ 0

PF3D7_0918600|PfBLM     ------------------------------------------------------------ 0
PF3D7_1429900|PfWRN     ------------------------------------------------------------ 0
NP_001274175.1|HsBLM    ttqntptvkksrdtalkklefssspdslstindwddmddfdt-------------setsk 167
NP_000544.2|HsWRN       -------------msekklet---taqqrkcpewmnvqnkrcaveerkacvrksvfeddl 44
DAA10088|ScSgs1         ---sgptn-----tatkqhe---vmqtlsndtewlsytatsn-------------qyadv 105
AAA24517|EcRecQ         ------------------------------------------------------------ 0

PF3D7_0918600|PfBLM     ------------------------------------------------------------ 0
PF3D7_1429900|PfWRN     ------------------------------------------------------------ 0
NP_001274175.1|HsBLM    sfvtppqshfvrv--staqkskkgkrnffkaqlytt---ntvktdlpppsseseqid--- 219
NP_000544.2|HsWRN       pfleftgsivysy--------dasdcsflsedismsl------------------sdgdv 78
DAA10088|ScSgs1         pmvdipastsvvsnprtpngskthnfntfrphmasslvendssrnlgsrnnnksvidnss 165
AAA24517|EcRecQ         ------------------------------------------------------------ 0

PF3D7_0918600|PfBLM     ------------------------------------------------------------ 0
PF3D7_1429900|PfWRN     ------------------------------------------------------------ 0
NP_001274175.1|HsBLM    -------------------lteeqkddsewlssdviciddgpi----------------- 243
NP_000544.2|HsWRN       vgfdm----ewpplynrgklgkvaliqlcvseskcylfhvssmsvfpqglkmllenkavk 134
DAA10088|ScSgs1         igkqlendiklevirlqgslimalkeqsklllqkcsiiestsls---------------- 209
AAA24517|EcRecQ         ------------------------------------------------------------ 0

PF3D7_0918600|PfBLM     ------------------------------------------------------------ 0
PF3D7_1429900|PfWRN     ------------------------------------------------------------ 0
NP_001274175.1|HsBLM    ---------------------aevhinedaqesdslkthlederdnsekkknleeaelhs 282
NP_000544.2|HsWRN       kagvgiegdqwkllrdfdiklknfveltdvank-klkctet-wslnslvkhllgkq-llk 191
DAA10088|ScSgs1         -----edakrlqlsrdirpqlsnmsiridslekeiikak----kdgmskdqskg------ 254
AAA24517|EcRecQ         ------------------------------------------------------------ 0

PF3D7_0918600|PfBLM     ------------------------------------------------------------ 0
PF3D7_1429900|PfWRN     ------------------------------------------------------------ 0
NP_001274175.1|HsBLM    tekvpciefdd------------ddydtdfvppspeeiisassssskc----lstlkdld 326
NP_000544.2|HsWRN       dksircsnwskfpltedqklyaatdayagfiiyrnlei---------------------- 229
DAA10088|ScSgs1         ---rsqvssqd------------dniis-silpspleyntssrnsnltsttattvtkala 298
AAA24517|EcRecQ         ------------------------------------------------------------ 0

PF3D7_0918600|PfBLM     ------------------------------------------------------------ 0
PF3D7_1429900|PfWRN     ------------------------------------------------------------ 0
NP_001274175.1|HsBLM    tsdrkedvlsts-kdl-lskpekmsmqelnpetstdcdarqislqqqlihvmehicklid 384
NP_000544.2|HsWRN       ---------lddtvqrfainkeeeillsdmnk--------------qltsiseevmdlak 266
DAA10088|ScSgs1         itgakqnitnntgknsnndsnnddliqvlddeddidcdppvilkegap-hs--------p 349
AAA24517|EcRecQ         ------------------------------------------------------------ 0

PF3D7_0918600|PfBLM     ------------------------------------------------------------ 0
PF3D7_1429900|PfWRN     ------------------------------------------------------------ 0
NP_001274175.1|HsBLM    tipddklklldcgnellqqrnirrklltevdfnksda----sllgslwr-yrpdsl---- 435
NP_000544.2|HsWRN       hlphafsklen----------prrvsillkdisenlyslrrmiigstnie---telrpsn 313
DAA10088|ScSgs1         afphlhm-tseeqdeltrrrnmrsrepvnyripdrddpf-dyvmgkslrddypdvereed 407
AAA24517|EcRecQ         ------------------------------------------------------------ 0

PF3D7_0918600|PfBLM     ------------------------------------------------------------ 0
PF3D7_1429900|PfWRN     ------------------------------------------------------------ 0
NP_001274175.1|HsBLM    dgpmeg-dscp-------------tgnsmkelnfshlpsns------------------- 462
NP_000544.2|HsWRN       nlnllsfedsttggv--------qqkqirehevlihvedetwdptldhlakhdgedvlgn 365
DAA10088|ScSgs1         eltmea-eddahssymttrdeekeenellnqsdfdfvvnddldptqdtd-yhdnmdvsan 465
AAA24517|EcRecQ         ------------------------------------------------------------ 0

PF3D7_0918600|PfBLM     ------------------------------------------------------------ 0
PF3D7_1429900|PfWRN     ------------------------------------------------------------ 0
NP_001274175.1|HsBLM    ----vspgdclltttl---------------------gktgfsatrknlferplfnthlq 497
NP_000544.2|HsWRN       kve-----------------------------------------rkedgfedgvednklk 384
DAA10088|ScSgs1         iqessqegdtrstitlsqnknvqvilssptaqsvpsnggnqigvehidlleddlekdail 525
AAA24517|EcRecQ         ------------------------------------------------------------ 0

PF3D7_0918600|PfBLM     ------------------------------------------------------------ 0
PF3D7_1429900|PfWRN     ------------------------------------------------------------ 0
NP_001274175.1|HsBLM    ks-----fvssnwa--etprlgkknessyfpgnvltstavkdqnkhtasindleretqps 550
NP_000544.2|HsWRN       enmeraclmslditehelqileqqsqeeylsdiay---------kstehlspndnendts 435
DAA10088|ScSgs1         dd-----smsfsf--------grqh----mpmshs-dlelidsekenedfeednnnngie 567
AAA24517|EcRecQ         ------------------------------------------------------------ 0

PF3D7_0918600|PfBLM     ---------------------------------------MNEDAMKIL------------ 9
PF3D7_1429900|PfWRN     ------------------------------------------------------------ 0
NP_001274175.1|HsBLM    ydidnfdiddfdddddwedimhnlaasksst--aayqpikegrpiksv--serlssaktd 606
NP_000544.2|HsWRN       yviesd------edlemem-lkhlspndnendtsyviesdedlememlkslenlnsgt-- 486
DAA10088|ScSgs1         -ylsdsdlerfdeerenrtqva------------diqeld--ndlkii--terkltgdne 610
AAA24517|EcRecQ         ------------------------------------------------------------ 0

PF3D7_0918600|PfBLM     --------------------NKCSGEKKKGE-----------DLNSCNLL-EYY-KTKY- 35
PF3D7_1429900|PfWRN     --------MLKFLNVKSPVVSSDVDEI--TD-------D------------SDKGDVSYT 31
NP_001274175.1|HsBLM    clp---------------v-sstaqninfsesiqnytdksaqnlasrnlkherfqslsfp 650
NP_000544.2|HsWRN       -vepthskclkmernlglp-tkeeeeddene----------------anegeedddkdfl 528
DAA10088|ScSgs1         hpppswspkik--rekssv-sqkdeeddfdddf------slsdivsksnlssktngptyp 661
AAA24517|EcRecQ         ----------------------------------------------mnv----aqaevln 10

PF3D7_0918600|PfBLM     SSIDIEKAKLILKEQFLIPDFREKQLECLNSIKRF-EHVLNIMPTGGGKSLIYQVLPLII 94
PF3D7_1429900|PfWRN     LEELRMKMEITQKKHFGYKNLKDFQVEAVHATFHK-KDSFIVMATGMGKSLCYQIPSLMD 90
NP_001274175.1|HsBLM    h---tkemmkifhkkfglhnfrtnqleainaallg-edcfilmptgggkslcyqlpacvs 706
NP_000544.2|HsWRN       wpapneeqvtclkmyfghssfkpvqwkvihsvleerrdnvavmatgygkslcfqyppvyv 588
DAA10088|ScSgs1         w---sdevlyrlhevfklpgfrpnqleavnatlqg-kdvfvlmptgggkslcyqlpavvk 717
AAA24517|EcRecQ         l---esgakqvlqetfgyqqfrpgqeeiidtvlsg-rdclvvmptgggkslcyqipalll 66
                                    :  *    ::  * : :.:     .. . :* ** **** :*

PF3D7_0918600|PfBLM     E----GISIVISPLISLIQDQIISLRNKKIVAETINSSLNKKENERILDILKSQDLGNLK 150
PF3D7_1429900|PfWRN     V-CRKKFTIVISPLISLMKDQVDNLNRKRISSVFLGSGQKMNNN----KILNEIKHGIYK 145
NP_001274175.1|HsBLM    p----gvtvvisplrslivdqvqkltsldipatyltgdktds-eatniylqlskkdpiik 761
NP_000544.2|HsWRN       g----kiglvisplislmedqvlqlkmsnipacflgsaqsen-v------ltdiklgkyr 637
DAA10088|ScSgs1         sgkthgttivisplislmqdqvehllnknikasmfssrgtae-qrrqtf--nlfinglld 774
AAA24517|EcRecQ         n----gltvvvsplislmkdqvdqlqangvaaaclnstqtre-qql--evmtgcrtgqir 119
                                :*:*** **: **:  *    : :  : .  . .
```

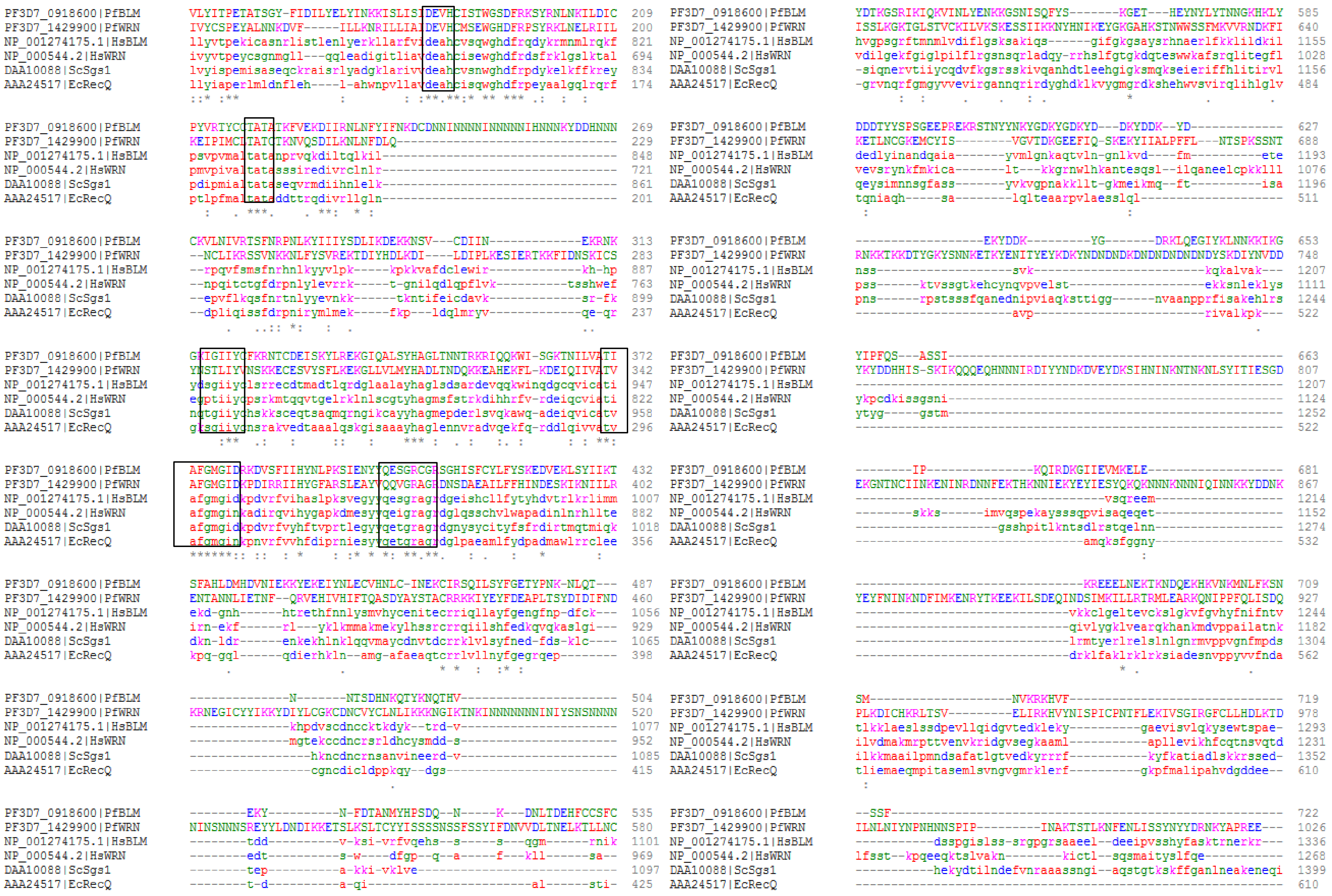
PF3D7_0918600|PfBLM
PF3D7_1429900|PfWRN
NP_001274175.1|HsBLM
NP_000544.2|HsWRN
DAA10088|ScSgs1
AAA24517|EcRecQ

```
PF3D7_0918600|PfBLM        -------KIPRKI-----------------------------------------------    728
PF3D7_1429900|PfWRN        -------------SK---------------------------------------------    1028
NP_001274175.1|HsBLM       ------kkmpasqrskr---rktassgskakggsatcrkissk----tksssiigsssa-    1382
NP_000544.2|HsWRN          ------kkmplksiaesrilplmtigmhlsqavkagcpldleragltpevqkiiadvirn    1322
DAA10088|ScSgs1            inqirqsqlpknttssksgtrsisksskksangrrgfrnyr-------------------    1440
AAA24517|EcRecQ            ------------------------------------------------------------    610

PF3D7_0918600|PfBLM        ------------------------------------------------------------    728
PF3D7_1429900|PfWRN        -------------------------------------------SNETYSYEHYTGTERKC    1045
NP_001274175.1|HsBLM       ----shts----------------------------------------------------    1386
NP_000544.2|HsWRN          ppvnsdmskislirmlvpenidtylihmaieilkhgpdsglqpscdvnkrrcfpgseeic    1382
DAA10088|ScSgs1            ------------------------------------------------------------    1440
AAA24517|EcRecQ            ------------------------------------------------------------    610

PF3D7_0918600|PfBLM        ------------------------------------------------------------    728
PF3D7_1429900|PfWRN        KHFVTQEDEYNHADISKMSRHTNIYEHKKNMNDHTYDLHNYNNNITYDYGDIPKNTLIQY    1105
NP_001274175.1|HsBLM       qatsgansklg------------imappkpinrp-flkpsya------------------    1415
NP_000544.2|HsWRN          ssskrskeevg------------intetssaerk-rrlpvwf-----akgsdtskkl---    1421
DAA10088|ScSgs1            ----------g------------hyrgrk-------------------------------    1447
AAA24517|EcRecQ            ------------------------------------------------------------    610

PF3D7_0918600|PfBLM        ------------------------------------------------------------    728
PF3D7_1429900|PfWRN        KNSLTDQNTISMSDINSDNKNMVYNNHIFNKNHSIITNYNSSAENNSSLQNEFYRKKTNI    1165
NP_001274175.1|HsBLM       ------------------------------------------------------------    1415
NP_000544.2|HsWRN          ------------------------------------------------------------    1421
DAA10088|ScSgs1            ------------------------------------------------------------    1447
AAA24517|EcRecQ            ------------------------------------------------------------    610

PF3D7_0918600|PfBLM        ------------------------------------------------------------    728
PF3D7_1429900|PfWRN        IDDNNIGHHTYNINDNYHTNDVLLTNAIIKDRPKKKHDDIFSNFCYKRDESETGVQGNVT    1225
NP_001274175.1|HsBLM       ----------------------------------------fs------------------    1417
NP_000544.2|HsWRN          m-------------------------------dktkrgglfs------------------    1432
DAA10088|ScSgs1            ------------------------------------------------------------    1447
AAA24517|EcRecQ            ------------------------------------------------------------    610

PF3D7_0918600|PfBLM        ------------------------------------------------------------    728
PF3D7_1429900|PfWRN        HVKKDINKGYQENKNDITFLQNNISQHTNNMIHNNGKDVKNNLSALDDDDDDDDDDFKFI    1285
NP_001274175.1|HsBLM       ------------------------------------------------------------    1417
NP_000544.2|HsWRN          ------------------------------------------------------------    1432
DAA10088|ScSgs1            ------------------------------------------------------------    1447
AAA24517|EcRecQ            ------------------------------------------------------------    610

PF3D7_0918600|PfBLM        ------------------------------------------------------------    728
PF3D7_1429900|PfWRN        NQIQTLGLHEQNCKEENNYQNSLTNEHVHIPNYNLYQEENTRNENEKRKININLKQEDQN    1345
NP_001274175.1|HsBLM       ------------------------------------------------------------    1417
NP_000544.2|HsWRN          ------------------------------------------------------------    1432
DAA10088|ScSgs1            ------------------------------------------------------------    1447
AAA24517|EcRecQ            ------------------------------------------------------------    610

PF3D7_0918600|PfBLM        ------------------------------------------------------------    728
PF3D7_1429900|PfWRN        IYTKRHQYLTNPNEHSVNNMHNIHLKENNIPKYDYTNQNVHESNQKKNDFYEEKKKKFNF    1405
NP_001274175.1|HsBLM       ------------------------------------------------------------    1417
NP_000544.2|HsWRN          ------------------------------------------------------------    1432
DAA10088|ScSgs1            ------------------------------------------------------------    1447
AAA24517|EcRecQ            ------------------------------------------------------------    610

PF3D7_0918600|PfBLM        -----------------------------------    728
PF3D7_1429900|PfWRN        IESFSYNNNQEDKKRVNNLDILDQFVYRDVKKRKT    1440
NP_001274175.1|HsBLM       -----------------------------------    1417
NP_000544.2|HsWRN          -----------------------------------    1432
DAA10088|ScSgs1            -----------------------------------    1447
AAA24517|EcRecQ            -----------------------------------    610
```
